# Error in Abstract

**DOI:** 10.1001/jamanetworkopen.2020.32624

**Published:** 2020-12-09

In the Original Investigation titled “Evaluation of Deep Learning to Augment Image-Guided Radiotherapy for Head and Neck and Prostate Cancers,”^1^ published November 30, 2020, there was an error in the Abstract. The sentence describing manual contouring times should have read “Manual segmentation of the head and neck took a mean 86.75 (95% CI, 75.21-92.29) min/scan for an expert reader and 73.25 (95% CI, 68.68-77.82) min/scan for a radiation oncologist. The autogenerated contours represented a 93% reduction in time.” This article has been corrected.^1^
